# Prevalence of rectovaginal colonization by group B *Streptococcus* in pregnant women seen at prenatal care program of a health organization

**DOI:** 10.31744/einstein_journal/2020AO4920

**Published:** 2019-12-03

**Authors:** Nilson Abrão Szylit, Fernanda Lima Malburg, Carla de Azevedo Piccinato, Lais Assenheimer de Paula Ferreira, Sérgio Podgaec, Eduardo Zlotnik

**Affiliations:** 1 Hospital Israelita Albert Einstein, São Paulo, SP, Brazil.

**Keywords:** Viridans streptococci, Prenatal care; Risk factors, Epidemiology, Pregnancy complications, infectious/diagnosis, Streptococcus agalactiae, Pregnancy, Prevalence

## Abstract

**Objective:**

To evaluate the prevalence of group B *Streptococci* in pregnant women of a corporate health program, as well as the epidemiological correlations.

**Methods:**

This retrospective study used medical records of patients who participated of the prenatal care program at a private hospital in the city of São Paulo (SP), Brazil, from 2015 to 2016. Those who abandoned the program or had incomplete data in their medical records were excluded. Quantitative variables were described by means, standard deviations, median, minimal and maximal values. Parity and socioeconomic status were described by absolute frequency and percentages. We used logistic regression models in the software (SPSS) to analyze correlations of variables according to vaginal-rectal culture, considering a 95%CI and p-values. Variables were age, number of pregnancies, weight gain in pregnancy and gestational age at delivery.

**Results:**

A total of 347 medical records were included, and after applying the exclusion criteria, 287 medical records composed the final sample. Patients’ age ranged between 17 and 44 years. Mean age was 30.6 years, 67 patients had positive result for group B *Streptococcus* (prevalence of 23.3%; 95%CI: 18.7-28.5).

**Conclusion:**

Considering the high prevalence of group B *Streptococcus* in our service, the antibiotic prophylaxis strategy based on rectovaginal culture screening approach seems to be cost-effective.

## INTRODUCTION

Group B hemolytic *Streptococcus* (GBS), or *Streptococcus agalactiae* , is a Gram-positive coccus that is part of the usual rectovaginal flora, and can be found transiently in asymptomatic women. ^1^ This bacterium is associated with early infection in newborns, and it is the leading cause of death in the neonatal period. ^2^ There are two clinical manifestations of the infection. Early-onset GBS disease (EOGBS), which appears up to the seventh day of life, accounts for 80% of cases of pneumonia, meningitis or sepsis. The late form manifests after the first week until the third month of life, and presents as meningitis in 24% of cases. ^3 , 4^

As from 1986, the Centers for Disease Control and Prevention (CDC) recommendation was to perform prophylactic antibiotic therapy (PAT) on pregnant women with risk factors. ^5^ Later on, in 2002, with the revision of the protocol, adopting the recommendation of performing universal screening of all women between 35 and 37 weeks of gestation using rectovaginal culture for identifying pregnant women colonized by GBS and administering PAT, the incidence of EOGBS was reduced by more than 80%. ^6 , 7^ In the United States, the reduction was from 1.8 cases in the early 1990’s to 0.23 cases per 1,000 live births, in 2015. PAT did not reduce the rate of late disease infection. ^4 , 6^

The prevalence of vaginal GBS colonization in pregnant women is 4% to 35%, which is similar to the female population in general. ^1 , 8 , 9^ Among pregnant women colonized by GBS, 50% to 75% of newborns exposed to GBS become colonized, and 1% to 2% of these infants develop EOGBS. ^1^ The mortality rate declined to 5% in 2014, a 91% decrease of the rate found 28 years ago. ^10 , 11^

The PAT-based prevention strategy of universal screening of all pregnant women has a higher cost than the strategy based on risk factors, and its efficacy has also been questioned, because it may increase bacterial resistance and the occurrence of EOGBS in newborn infants of pregnant women with GBS-negative culture in antepartum screening. ^4 , 12^ Knowledge of GBS colonization prevalence in pregnant women should be considered for the implementation of an appropriate strategy for EOGBS prevention, which is different in several population groups. ^9^

The study of the cost-effectiveness of prenatal screening and test programs crucially depends on the values attributed to the adverse outcomes prevented by the test and requires explicit public debate, bearing in mind the long-term consequences for the children who survive GBS infection. ^13^

## OBJECTIVE

To identify the prevalence of group B streptococcal colonization among pregnant women, and to evaluate its association with age, parity, and gestational age at delivery.

## METHODS

A retrospective study was conducted in the medical records of the Healthy Pregnancy Program, which serves employees and their dependents of *Hospital Israelita Albert Einstein* (HIAE), in the city of São Paulo (SP), from March 2015 to December 2016. The study was approved by the local Research Ethics Committee (CAAE: 65896717.6.0000.00071; opinion number: 1.999.826).

All 347 medical records of pregnant women attended by the program were included for manual review. Of these, 60 were excluded: 31 patients did not complete prenatal care and 29 did not have sufficient data regarding rectovaginal GBS culture test results. From the remaining 287 patients, the following characteristics were collected: age, position held, schooling level, number of pregnancies, body mass index (BMI), weight gain during pregnancy, gestational age (GA) at delivery, and rectovaginal GBS culture test results. The data were separated to be analyzed in two groups according to vaginal and anal GBS culture test results, performed between 35 and 37 weeks of gestation. To calculate the BMI, the weight of the pregnant woman was considered as the weight measured in the first medical visit recorded in the medical record. To calculate weight gain during pregnancy, the final weight was considered as the weight measured at the last medical visit. To determine the socioeconomic status, patients were divided into two classes according to the level of education required for the function: (Class 1: women whose position required complete higher education; Class 2: women who held positions that required complete high school or technical education only).

Quantitative variables were described by means, standard deviations, medians, minimum and maximum values. Parity and economic class were described by absolute frequencies and percentages. ^14^ The investigation of the factors affecting the positivity was conducted using logistic regression models, ^15^ and the results were presented by odds ratios, 95% confidence intervals (95%CI), and p values. The analyses were performed using the software (SPSS), version 24.0. ^16^

## RESULTS

Data from 287 pregnant women aged 17 to 44 years were analyzed. Of these patients, 67 had a positive result for GBS, which corresponded to a prevalence of 23.3% (95%CI: 18.7-28.5). The characteristics of the patients, divided according to the GBS culture test results, are shown in table 1 . Gestational age at delivery varied between 34 and 41 weeks. Two cases with GA below 36 weeks (1 at 34 weeks, and 1 at 35 weeks) were observed.

**Table 1:** Data of pregnant women evaluated according to group B *Streptococcal* culture test results

	Group B hemolytic *Streptococcus*		

	No (220)	Yes (67)	Total (287)
Parity (number of previous deliveries) n (%)			
1	92 (42.0)	30 (44.8)	122 (42.7)
2	79 (36.1)	24 (35.8)	103 (36.0)
3	48 (21.9)	13 (19.4)	61 (21.3)
Total	219 (100.0)	67 (100.0)	286 (100.0)
Socioeconomic level n (%)			
Class 1	82 (42.3)	28 (46.7)	110 (43.3)
Class 2	112 (57.7)	32 (53.3)	144 (56.7)
Total	194 (100.0)	60 (100.0)	254 (100.0)
Age (years)			
Mean (standard deviation)	30.7 (4.7)	30.7 (5.5)	30.7 (4.9)
Median (1 ^st^ quartile - 3 ^rd^ quartile)	31.0 (28.0; 34.0)	31.0 (26.0; 35.0)	31.0 (27.0; 35.0)
Minimum - Maximum	17 - 40	17 - 44	17 - 44
	n=220	n=67	n=287
Mother’s initial weight in first visit			
Mean (standard deviation)	68.1 (13.2)	68.3 (11.0)	68.1 (12.7)
Median (1 ^st^ quartile - 3 ^rd^ quartile)	66.0 (58.5; 75.0)	68.0 (61.0; 76.0)	67.0 (59.0; 75.0)
Minimum - Maximum	40 - 114	47 - 99	40 - 114
	n=216	n=67	n=283
Body mass index			
Mean (standard deviation)	25.7 (4.7)	25.4 (4.0)	25.7 (4.6)
Median (1 ^st^ quartile - 3 ^rd^ quartile)	24.8 (22.4; 28.0)	25.0 (22.4; 27.6)	25.0 (22.4; 27.8)
Minimum - Maximum	17 - 43	19 - 39	17 - 43
	n=179	n=53	n=232
Final weight (last visit before delivery)			
Mean (standard deviation)	79.5 (12.6)	80.3 (11.7)	79.7 (12.4)
Median (1 ^st^ quartile - 3 ^rd^ quartile)	77.0 (71.0; 87.0)	79.0 (72.0; 87.0)	77.0 (72.0; 87.0)
Minimum - Maximum	55 - 122	59 - 107	55 - 122
	n=212	n=62	n=274
Weight gain during pregnancy (in kg)			
Mean (standard deviation)	11.6 (4.1)	12.2 (5.1)	11.7 (4.4)
Median (1 ^st^ quartile - 3 ^rd^ quartile)	11.0 (8.0; 14.0)	12.0 (8.0; 15.0)	11.0 (8.0; 14.0)
Minimum - Maximum	2 - 24	2 - 24	2 - 24
	n=209	n=62	n=271
Gestational age			
Mean (standard deviation)	38.7 (1.0)	38.8 (0.8)	38.7 (1.0)
Median (1 ^st^ quartile - 3 ^rd^ quartile)	39.0 (38.0; 39.0)	39.0 (38.0; 39.0)	39.0 (38.0; 39.0)
Minimum - Maximum	34 - 41	36 - 40	34 - 41
	n=210	n=66	n=276

The relation between the variables of interest and positive GBS was studied using simple logistic regression models, which considered one explanatory variable at a time, and by multiple model, including all variables at the same time. No evidence of association with GBS was found ( Table 2 ).

**Table 2:** Regression models for explaining positivity for group B *Streptococcus*

	Simple model		Multiple model	
	
	Odds ratio (95%CI)	p value	Odds ratio (95%CI)	p value
Parity				
1	Reference		Reference	
2	0.932 (0.504; 1.724)	0.822	1.127 (0.525; 2.420)	0.759
3	0.831 (0.397; 1.738)	0.622	0.726 (0.271; 1.945)	0.524
Socioeconomic level				
Class 1	Reference		Reference	
Class 2	0.837 (0.468; 1.497)	0.548	0.892 (0.432; 1.841)	0.756
Age (years)	1.002 (0.948; 1.060)	0.944	1.011 (0.934; 1.095)	0.781
Body mass index	0.983 (0.917; 1.053)	0.624	0.971 (0.891; 1.058)	0.499
Weight gain in pregnancy (in kg)	1.034 (0.969; 1.103)	0.311	1.006 (0.926; 1.093)	0.882
Gestational age	1.115 (0.831; 1.496)	0.467	1.035 (0.738; 1.451)	0.843

95%CI: 95% confidence interval.

There was no difference in the mean age of pregnant women and gestational age at delivery between the positive and negative GBS groups (p>0.05). Likewise, the distribution of negative and positive patients according to parity, professional category and weight profile did not differ significantly between groups. Table 3 compares the prevalence found in this study with the prevalence in other regions of Brazil.

**Table 3:** Prevalence of group B *Streptococci* during pregnancy in several Brazilian locations (8,17-23)

Author	Local	Prevalence (%)
Linhares et al. ^(8)^	Ceará	4.2
Zusman et al. ^(17)^	Ribeirão Preto	17.9
Rocchetti et al ^.(18)^	São Paulo	25.4
Soares et al. ^(19)^	Rio de Janeiro	24.3
Wollheim et al. ^(20)^	Caxias do Sul	22.5-26*
Freitas et al. ^(21)^	Brasília	24.0
Gouvea et al. ^(22)^	Rio de Janeiro	19.04
Castellano-Filho et al. ^(23)^	Juiz de Fora	32.6
Results of this study	São Paulo	23.3

* Depending on laboratory culture and PCR methods, respectively.

Multiple model adjusted with 197 observations. In simple models, the number of observations ranged from 232 (body mass index) to 287 (age).

## DISCUSSION

This study found a GBS prevalence of 23.3% in the population of pregnant women followed by an HIAE antenatal care program, *i.e* ., higher than the world average, but similar to the average rates found in current Brazilian studies (4.2 to 32.6%). ^8 , 17 - 23^ A meta-analysis published in 2016 estimated that the overall prevalence of pregnant women with GBS is approximately 17.9% (95%CI: 16.2-19.7), with 11.1% (95%CI: 6.8-15.3) in Southeast Asia, 22.4% in Africa (95%CI: 18.1-26.7), 19% in Europe (95%CI: 16.1-22.0), and 19.7% in the American continent (95%CI: 16.7-22.7). ^24^

The variation in the prevalence rates may occur due to test technique, collection logistics, and delivery to the laboratory, ^25^ but the large variation in the rate cannot be explained only by the methodology used for the culture, the moment when the collection is performed during pregnancy, or the sample size. ^24^ This may be due to regional characteristics, ethnic diversity, temperature, and diet. ^17 , 23^

There was no association among socioeconomic, weight or demographic risk factors and vaginal GBS colonization in the population evaluated in our study. In the professional category classes 1 and 2, it is likely that the members of the groups had better economic status according to the position they held, but in class 3, which comprised their dependents, this analysis was not possible.

Similar data were presented in an 8-year study of 3,647 pregnant women in Rio de Janeiro, ^26^ and in a meta-analysis that analyzed 73,791 pregnant women, in 37 countries. ^17^ Also a study conducted in the city of Ribeirão Preto, comparing epidemiological data of pregnant women (n=249), with different socioeconomic profiles, colonized by GBS and treated at two hospitals found no relation with this factor. ^17^ Two American studies detected a higher incidence of EOGBS in the black population as compared to the white population. The first study found twice the rate of EOGBS (relative risk - RR=4.0; 95%CI: 2.9-5.5) and invasive GBS infection in pregnant women (RR=5.0; 95%CI: 2.9-8.7). ^27^ No explanation was found for this finding, but the database studied did not have socioeconomic information, which precludes the study of this correlation. ^28^ In the second study, birth records were analyzed and, after adjusting for confounding factors, increased maternal colonization by GBS was associated with black race (odds ratio — OR=1.54; 95%CI: 1.36-1.74) ^29^ Zusman et al., found no racial differences in Brazil and attributed this result to the population miscegenation found in the country. ^17^ However, there are reports of a positive association between prevalence and some of the factors evaluated. For example, there was a positive association between GBS colonization and increased age of pregnant women. ^30 , 31^ On the other hand, an American study of 59,965 pregnant women identified a lower colonization rate in younger women (RR=0.99; 95%CI: 0.99-0.99). ^1^

Obesity is not generally considered a risk factor for GBS colonization. However, another American study indicated an association with an increase in patients’ BMI. The reason for this relation was unclear and may be linked to changes in the gastrointestinal microbiota in obese women. ^32^ Regarding parity, the literature presents conflicting data. Whereas a study indicates a higher prevalence in the first pregnancy, ^33^ another study reports an increase in prevalence after the fifth pregnancy. ^31^ There are no data in these publications to exclude confounding factors, making interpretation of the findings difficult. Other populations studied showed no relation with parity. ^34 , 35^

Without the use of PAT, approximately 50% of newborns of pregnant women colonized by GBS are infected at birth, and the incidence of EOGBS in this group is 30 times higher than in newborns of pregnant women in whom the GBS culture test was negative. ^36^ PAT, based on universal screening of all pregnant women by rectovaginal culture, between 35 and 37 weeks of gestation, reduced the incidence in the United States by 80%. ^36^ On the other hand, the Royal College of Obstetricians and Gynaecologists (RCOG) recommends that prevention by universal screening between 35 and 37 weeks should not be done, ^12^ a practice also adopted in Denmark, the Netherlands, and Australia. ^12 , 37^ Its advocates argue that the strategy based on universal screening has a higher cost than the strategy based on risk factors. The arguments for this position are: 17% to 25% of women who are screened positive for GBS between 35 and 37 weeks will not be colonized at delivery and, on the other hand, 5% to 7% of women with prenatal negative cultures will be colonized at delivery. ^12 , 37^ Prevention by universal screening has also been challenged by the possibility of increasing bacterial resistance by using PAT in a large number of women, ^12^ possible anaphylactic reactions, and alleged delayed metabolic and immunity alterations, which could be caused by alteration of the intestinal microbiome in the children of parturient women who received PAT. ^38^

Some GBS vaccines are under development and, when available, should avoid the need for PAT. ^25^ The motivation for this study was the fact that assessing the prevalence of maternal colonization by GBS is important for the definition of the best strategy in the prevention of EOGBS, which becomes cost effective when the prevalence is greater than 10%. ^39 , 40^ The efficiency of the adopted model depends on the implementation and adherence to a national protocol, which can be adapted to regional or even local particularities. ^41^ Ideally, this model should target a specific population, directing the treatment to women at risk and avoiding unnecessary interventions and costs. ^41^

The present study has limitations because it is retrospective, and the population evaluated is specific of a single antenatal care service, and the results cannot be extrapolated to the general population. However, for the establishment of a PAT protocol in the service at issue, it is important that we have this information. Other factors that should be considered, such as the number of documented cases of infection, were not analyzed. In addition, in this organization, the number of premature births, in which the highest incidence of EOGBS occurs, is lower than the global rate reported, which may reflect the absence of culture test results at delivery or the loss of this information.

## CONCLUSION

The prevalence found in the pregnant women evaluated in this study was higher than the world average, which indicates that there is cost-effectiveness in selecting those who should undergo prophylactic antibiotic therapy. Further analysis of other factors related to this protocol is required.
