# Supplementary material for: Prevalence of rectovaginal colonization by group B Streptococcus in pregnant women seen at prenatal care program of a health organization
Source: Einstein (Sao Paulo). 2019 Dec 3;18:eAO4920. doi: 10.31744/einstein_journal/2020AO4920 (PMC6905166; doi:10.31744/einstein_journal/2020AO4920)
Supplement: Translation

# Translation: Prevalência de colonização retovaginal por estreptococo do grupo B em gestantes de programa de atendimento pré-natal de instituição de saúde

**Authors:** Nilson Abrão Szylit, Fernanda Lima Malburg, Carla de Azevedo Piccinato, Lais Assenheimer de Paula Ferreira, Sérgio Podgaec, Eduardo Zlotnik

**Affiliations:** 1 Hospital Israelita Albert Einstein, São Paulo, SP, Brasil .

**Keywords:** Estreptococos viridans, Cuidado pré-natal, Fatores de risco, Epidemiologia, Complicações infecciosas na gravidez/diagnóstico, Streptococcus agalactiae, Gravidez, Prevalência

## Abstract

**Objetivo:**

Identificar a prevalência de estreptococo do grupo B entre gestantes que frequentaram um programa de saúde corporativa, bem como as correlações com a colonização positiva.

**Métodos:**

Estudo retrospectivo dos prontuários do pré-natal de um hospital privado em São Paulo, no período de 2015 a 2016. Foram excluídas as mulheres que abandonaram o programa ou apresentavam dados incompletos nos prontuários. As variáveis quantitativas foram descritas por média, desvios padrão, mediana, valores mínimos e máximos. A paridade e a condição socioeconômica foram descritos por frequência absoluta e percentagens. Utilizamos modelos de regressão logística no programa (SPSS) para analisar as correlações de variáveis de acordo com a cultura retovaginal, considerando IC95% e valores de p. As variáveis foram idade, número de gestações, peso ganho na gestação e idade gestacional no parto.

**Resultados:**

Foram incluídos 347 prontuários e, após a aplicação dos critérios de exclusão, 287 prontuários compuseram a amostra final. A idade dos pacientes variou entre 17 e 44 anos. A média de idade foi de 30,6 anos, e 67 pacientes tiveram resultado positivo para o estreptococo do grupo B (prevalência de 23,3%; IC95%: 18,7-28,5).

**Conclusão:**

Considerando a alta prevalência de estreptococos do grupo B em nosso serviço, existem evidências de que a estratégia de antibiótico profilaxia baseada na cultura retovaginal é custo-efetiva.

## INTRODUÇÃO

Estreptococo hemolítico do grupo B (GBS), ou *Streptococcus agalactiae* , é um coco *Gram* -positivo que faz parte da flora retovaginal habitual, e pode ser encontrado de forma transitória em mulheres assintomáticas.^1^Esta bactéria está associada à infecção precoce em recém-nascidos, sendo a principal causa de morte no período neonatal.^2^Existem duas formas clínicas de manifestação da infecção. A doença invasiva precoce pelo GBS (EOGBS - *early-onset* GBS *disease* ), que aparece até o sétimo dia de vida, sendo responsável por 80% das ocorrências na forma de pneumonia, meningite ou septicemia. A forma tardia se manifesta após a primeira semana até o terceiro mês de vida e aparece sob a forma de meningite em 24% dos casos.^3 , 4^

A recomendação do *Centers for Disease Control and Prevention* (CDC) é de realizar antibioticoterapia profilática (ATBP) em gestantes com fatores de risco, a partir de 1986.^5^Posteriormente, em 2002, com a revisão do protocolo, adotando a recomendação da triagem universal por meio da cultura retovaginal de todas as mulheres entre 35 e 37 semanas de gestação para identificação e ATBP das grávidas colonizadas pelo GBS, a incidência da EOGBS foi reduzida em mais de 80%.^6 , 7^Nos Estados Unidos, a redução foi de 1,8 caso no início de 1990 para 0,23 caso por 1.000 nascidos vivos em 2015. A ATBP não reduziu a taxa de infecção da doença tardia.^4 , 6^

A prevalência da colonização vaginal do GBS nas gestantes é de 4% a 35%, valor este semelhante ao da população feminina em geral.^1 , 8 , 9^Dentre as gestantes colonizadas pelo GBS, 50% a 75% dos recém-nascidos expostos ao GBS tornam-se colonizados, sendo que 1% a 2% destes bebês desenvolverão a EOGBS.^1^A taxa de mortalidade declinou para 5%, em 2014, diminuindo 91% em relação à taxa encontrada há 28 anos.^10 , 11^

A estratégia de prevenção baseada na ATBP pela triagem universal de todas as gestantes, apresenta custo maior do que a baseada nos fatores de risco e, também tem sido questionada por poder aumentar a resistência bacteriana, bem como pela ocorrência de EOGBS em recém-nascidos de gestantes com cultura negativa para o GBS na triagem anteparto.^4 , 12^O conhecimento da prevalência da colonização de gestantes pelo GBS deve ser considerado para a implementação de estratégia adequada para a prevenção da EOGBS, sendo diferente em diversos grupos populacionais.^9^

O estudo de custo-benefício de programas de triagem e teste pré-natal depende crucialmente dos valores atribuídos aos resultados adversos evitados pelo exame e necessitam de debates públicos explícitos, tendo em mente as consequências a longo prazo das crianças que sobrevivem à infecção causada pelo GBS.^13^

## OBJETIVO

Identificar a prevalência da colonização por estreptococo do grupo B entre gestantes, assim como avaliar sua associação com idade, paridade e idade gestacional no parto.

## MÉTODOS

Foi realizado estudo retrospectivo nos prontuários das pacientes do Programa Gestação Saudável, que atende funcionários e dependentes do Hospital Israelita Albert Einstein (HIAE), na cidade de São Paulo (SP), no período compreendido entre março de 2015 a dezembro de 2016. O estudo foi aprovado pelo Comitê de Ética em pesquisa da instituição (CAAE: 65896717.6.0000.00071; parecer: 1.999.826).

Foram incluídos, para revisão manual, todos os 347 prontuários das gestantes atendidas pelo programa. Destes, 60 foram excluídos: 31 pacientes não concluíram o pré-natal e 29 não apresentavam dados suficientes relativos ao resultado da cultura retovaginal para o GBS. Das 287 pacientes restantes, foram coletadas as seguintes características: idade, cargo ocupado, nível educacional, número de gestações, índice de massa corporal (IMC), ganho de peso na gestação, idade gestacional (IG) no momento do parto e resultado da cultura retovaginal para o GBS. Os dados foram separados para serem analisados em dois grupos, de acordo com o resultado da cultura vaginal e anal para o GBS, realizada entre a 35ª e a 37ª semana de gestação. Para o cálculo do IMC, foi considerado o peso da gestante na primeira consulta anotado no prontuário. No cálculo de ganho de peso durante a gestação, o peso final foi considerado como o da última consulta. Para a determinação da condição socioeconômica, as pacientes foram divididas em duas classes de acordo com o nível educacional necessário para a função: (Classe 1: mulheres que necessitavam de Ensino Superior completo para atuar no cargo; Classe 2: mulheres que possuíam cargos que exigiam apenas Ensino Médio completo ou ensino técnico).

As variáveis quantitativas foram descritas por médias, desvios padrão, mediana, e valores mínimo e máximo. Paridade e classe econômica foram descritas por frequências absolutas e percentagens.^14^A investigação dos fatores associados à positividade foi feita por modelos de regressão logística,^15^sendo os resultados apresentados por razões de chances, intervalos de confiança de 95% (IC95%) e valores de p. As análises foram realizadas com o auxílio do programa (SPSS), versão 24.0.^16^

## RESULTADOS

Foram analisados os dados de 287 gestantes com idade variando entre 17 e 44 anos. Dessas pacientes analisadas, 67 apresentaram resultado positivo para GBS, o que correspondeu a prevalência de 23,3% (IC95%: 18,7-28,5). As características das pacientes, divididas de acordo com o resultado da cultura para o GBS, estão expostas na tabela 1 . A IG na ocasião do parto variou entre 34 e 41 semanas. Foram observados dois casos com IG abaixo de 36 semanas (1 com 34 e 1 com 35 semanas).

**Tabela 1:** Dados das gestantes avaliadas, de acordo com o resultado da cultura para estreptococo do grupo B

	Estreptococo hemolítico do grupo B		

	Não (220)	Sim (67)	Total (287)
Paridade (Quantos partos anteriores) n (%)			
1	92 (42,0)	30 (44,8)	122 (42,7)
2	79 (36,1)	24 (35,8)	103 (36,0)
3	48 (21,9)	13 (19,4)	61 (21,3)
Total	219 (100,0)	67 (100,0)	286 (100,0)
Nível socioeconômico n (%)			
Classe 1	82 (42,3)	28 (46,7)	110 (43,3)
Classe 2	112 (57,7)	32 (53,3)	144 (56,7)
Total	194 (100,0)	60 (100,0)	254 (100,0)
Idade (anos)			
Média (desvio-padrão)	30,7 (4,7)	30,7 (5,5)	30,7 (4,9)
Mediana (1º quartil-3º quartil)	31,0 (28,0; 34,0)	31,0 (26,0; 35,0)	31,0 (27,0; 35,0)
Mínimo - Máximo	17 - 40	17 - 44	17 - 44
	n=220	n=67	n=287
Peso inicial da mãe na primeira consulta			
Média (desvio-padrão)	68,1 (13,2)	68,3 (11,0)	68,1 (12,7)
Mediana (1º quartil-3º quartil)	66,0 (58,5; 75,0)	68,0 (61,0; 76,0)	67,0 (59,0; 75,0)
Mínimo - Máximo	40 - 114	47 - 99	40 - 114
	n=216	n=67	n=283
Índice de massa corpórea			
Média (desvio-padrão)	25,7 (4,7)	25,4 (4,0)	25,7 (4,6)
Mediana (1º quartil-3º quartil)	24,8 (22,4; 28,0)	25,0 (22,4; 27,6)	25,0 (22,4; 27,8)
Mínimo - Máximo	17 - 43	19 - 39	17 - 43
	n=179	n=53	n=232
Peso final (última consulta antes do parto)			
Média (desvio-padrão)	79,5 (12,6)	80,3 (11,7)	79,7 (12,4)
Mediana (1º quartil-3º quartil)	77,0 (71,0; 87,0)	79,0 (72,0; 87,0)	77,0 (72,0; 87,0)
Mínimo - Máximo	55 - 122	59 - 107	55 - 122
	n=212	n=62	n=274
Ganho de peso na gestação (em kg)			
Média (desvio-padrão)	11,6 (4,1)	12,2 (5,1)	11,7 (4,4)
Mediana (1º quartil-3º quartil)	11,0 (8,0; 14,0)	12,0 (8,0; 15,0)	11,0 (8,0; 14,0)
Mínimo - Máximo	2 - 24	2 - 24	2 - 24
	n=209	n=62	n=271
Idade gestacional			
Média (desvio-padrão)	38,7 (1,0)	38,8 (0,8)	38,7 (1,0)
Mediana (1º quartil-3º quartil)	39,0 (38,0; 39,0)	39,0 (38,0; 39,0)	39,0 (38,0; 39,0)
Mínimo - Máximo	34 - 41	36 - 40	34 - 41
	n=210	n=66	n=276

Foi estudada a relação entre as variáveis de interesse e a positividade para GBS por meio de modelos de regressão logística simples, que consideraram uma variável explicativa de cada vez, e por modelo múltiplo, incluindo todas as variáveis ao mesmo tempo, não tendo sido encontradas evidências de associação com GBS ( Tabela 2 ).

**Tabela 2:** Modelos de regressão para a explicação de positividade para estreptococo do grupo B

	Modelos simples		Modelo múltiplo	
	
	Razão de chances (IC95%)	Valor de p	Razão de chances (IC95%)	Valor de p
Paridade				
1	Referência		Referência	
2	0,932 (0,504; 1,724)	0,822	1,127 (0,525; 2,420)	0,759
3	0,831 (0,397; 1,738)	0,622	0,726 (0,271; 1,945)	0,524
Nível socioeconômico				
Classe 1	Referência		Referência	
Classe 2	0,837 (0,468; 1,497)	0,548	0,892 (0,432; 1,841)	0,756
Idade (anos)	1,002 (0,948; 1,060)	0,944	1,011 (0,934; 1,095)	0,781
Índice de massa corpórea	0,983 (0,917; 1,053)	0,624	0,971 (0,891; 1,058)	0,499
Ganho de peso na gestação (em kg)	1,034 (0,969; 1,103)	0,311	1,006 (0,926; 1,093)	0,882
Idade gestacional	1,115 (0,831; 1,496)	0,467	1,035 (0,738; 1,451)	0,843

IC95%: intervalo de confiança de 95%.

Não houve diferença das médias de idade da gestante e IG ao nascimento entre os grupos GBS positivo e negativo (p>0,05). Da mesma forma, a distribuição de pacientes negativas e positivas, de acordo com paridade, categoria profissional e perfil ponderal, não apresentou diferença significativa entre os grupos. Tabela 3 compara a prevalência encontrada com a de outras regiões do Brasil.

**Tabela 3:** Prevalência de estreptococos do grupo B na gestação em várias localidades brasileiras (8,17-23)

Autor	Local	Prevalência (%)
Linhares et al.^(8)^	Ceará	4,2
Zusman et al.^(17)^	Ribeirão Preto	17,9
Rocchetti et al^.(18)^	São Paulo	25,4
Soares et al.^(19)^	Rio de Janeiro	24,3
Wollheim et al.^(20)^	Caxias do Sul	22,5-26*
Freitas et al.^(21)^	Brasília	24,0
Gouvea et al.^(22)^	Rio de Janeiro	19,04
Castellano-Filho et al.^(23)^	Juiz de Fora	32,6
Resultados do presente estudo	São Paulo	23,3

* Dependendo do método laboratorial cultura e PCR, respectivamente.

Modelo múltiplo ajustado com 197 observações. Nos modelos simples o número de observações variou entre 232 (índice de massa corpórea) e 287 (idade).

## DISCUSSÃO

Este estudo encontrou prevalência de 23,3% do GBS na população de gestantes seguidas em programa de atendimento pré-natal do HIAE, ou seja, maior do que a média mundial, porém semelhante às taxas médias encontradas em estudos brasileiros atuais (4,2 a 32,6%).^8 , 17 - 23^Metanálise publicada em 2016 estimou que a prevalência global de gestantes portadoras do GBS é de aproximadamente 17,9% (IC95%: 16,2-19,7), sendo no sudeste da Ásia de 11,1% (IC95%: 6,8-15,3), na África de 22,4% (IC95%: 18,1-26,7), na Europa de 19% (IC95%: 16,1-22,0) e no continente americano de 19,7% (IC95%: 16,7-22,7).^24^

A variação nas taxas de prevalência pode ocorrer pela técnica do exame, pela logística de coleta e pelo envio para o laboratório,^25^mas a grande variação da taxa não pode ser explicada apenas pela metodologia utilizada para a cultura, pelo momento em que a coleta é realizada durante a gestação ou pelo tamanho da amostra.^24^Ela pode ser decorrente de características regionais, da diversidade étnica, da temperatura e da alimentação.^17 , 23^

Não houve associação entre fatores de risco socioeconômicos, ponderais ou demográficos e a colonização vaginal pelo GBS na população avaliada em nosso estudo. Entre as classes 1 e 2, referentes às categorias profissionais, é provável que as integrantes do grupo possuíam condições econômicas melhores pelo cargo que ocupavam, mas, na classe 3, que é de dependentes, essa análise não foi possível.

Dados semelhantes foram apresentados em um estudo ao longo de 8 anos com 3.647 gestantes no Rio de Janeiro,^26^e em metanálise que analisou 73.791 gestantes, em 37 países.^17^Também um estudo realizado em Ribeirão Preto, comparando os dados epidemiológicos de gestantes (n=249) colonizadas pelo GBS e atendidas em dois hospitais, com perfis socioeconômicos diferentes, não encontrou relação com este fator.^17^Dois estudos americanos detectaram maior incidência da EOGBS, na população negra em relação à branca. O primeiro encontrou o dobro da taxa de EOGBS (risco relativo: RR=4,0; IC95%: 2,9-5,5) e de infecção invasiva pelo GBS nas gestantes (RR=5,0; IC95%: 2,9-8,7).^27^Não foi encontrada explicação para esse achado, mas o banco de dados estudado não possuía informações socioeconômicas, impedindo o estudo dessa correlação.^28^No segundo, foram analisados os registros de nascimentos e, após ajuste para os fatores de confusão, o aumento da colonização materna pelo GBS esteve associado com a raça negra (razão de chances - RC=1,54; IC95%: 1,36-1,74).^29^Zusman et al., não encontraram em nosso meio diferenças raciais e atribuíram este resultado à miscigenação populacional encontrada no país.^17^Porém, existem relatos de associação positiva entre a prevalência e alguns destes fatores avaliados. Por exemplo, houve associação positiva entre colonização por GBS e aumento da idade da gestante.^30 , 31^Por outro lado, estudo americano com 59.965 gestantes identificou taxa de colonização menor nas mulheres mais jovens (RR=0,99; IC95%: 0,99-0,99).^1^

A obesidade não é considerada, em geral, um fator de risco relacionado à colonização do GBS. Todavia, outro estudo americano indicou a associação com o aumento do IMC das pacientes. O motivo dessa relação não foi claro, podendo estar ligado às alterações da microbiota gastrintestinal nas mulheres obesas.^32^Em relação à paridade, a literatura apresenta dados conflitantes. Enquanto estudo aponta para prevalência maior na primeira gestação,^33^outro refere aumento da prevalência após a quinta gravidez.^31^Não existem, nestas publicações, dados que permitam excluir os fatores de confusão, dificultando a interpretação dos achados. Outras populações estudadas não mostraram relação com a paridade.^34 , 35^

Sem a utilização da ATBP, aproximadamente 50% dos recém-nascidos de gestantes colonizadas pelo GBS são infectados no momento do nascimento, sendo a incidência de EOGBS, neste grupo, 30 vezes maior do que nos recém-nascidos de gestantes nas quais a cultura foi negativa para o GBS.^36^A ATBP, baseada na triagem universal de todas as gestantes pela cultura retovaginal, entre 35 e 37 semanas de gestação, reduziu a incidência nos Estados Unidos em 80%.^36^Por outro lado, o *Royal College of Obstetricians and Gynaecologists* (RCOG) recomenda que a prevenção pelo rastreamento universal entre 35 e 37 semanas não deve ser feita,^12^conduta também adotada na Dinamarca, na Holanda e Austrália.^12 , 37^Seus defensores alegam que a estratégia baseada na triagem universal tem custo maior, em relação à baseada nos fatores de risco. Os argumentos para essa posição são: 17% a 25% das mulheres que têm o resultado da triagem positivo para o GBS entre 35 e 37 semanas não estarão colonizadas no momento do parto e, por outro lado, 5% a 7% das mulheres com cultura negativa no pré-natal estarão colonizadas no momento do parto.^12 , 37^A prevenção pela triagem universal também tem sido contestada pela possibilidade de aumentar a resistência bacteriana pela utilização de ATBP em um grande número de mulheres,^12^e pelas possíveis reações anafiláticas e supostas alterações metabólicas e de imunidade tardias, que poderiam ser causadas pela alteração do microbioma intestinal nas crianças de parturientes que receberam a ATBP.^38^

Algumas vacinas contra o GBS se encontram em desenvolvimento e, quando estiverem disponíveis, devem evitar a necessidade da ATBP.^25^A motivação para a realização deste estudo foi o fato que a avaliação da prevalência da colonização materna pelo GBS é importante para a definição da melhor estratégia na prevenção da EOGBS, passando a ser custo efetiva quando a prevalência for maior do que 10%.^39 , 40^A eficiência do modelo adotado depende da implantação e da aderência a um protocolo nacional, que possa ser adaptado às particularidade regionais e até de hospitais.^41^O ideal é que esse modelo esteja voltado para uma população específica, direcionando o tratamento para mulheres que apresentam risco e evitando intervenções e custos desnecessários.^41^

O presente estudo apresenta limitações, por ser retrospectivo e a população avaliada ser específica de um único serviço de pré-natal, não podendo o resultado ser extrapolado para a população em geral. No entanto, para o estabelecimento de um protocolo para a ATBP no serviço em questão é importante dispormos dessa informação. Outros fatores que devem ser considerados, como, por exemplo, número de casos documentados de infecção, não foram analisados. Além disso, na instituição, o número de partos prematuros, nos quais ocorre a maior incidência de EOGBS, é menor do que a taxa mundial referida, podendo refletir a ausência do resultado da cultura no momento do parto ou a perda dessa informação.

## CONCLUSÃO

A prevalência encontrada nas gestantes avaliadas neste estudo foi mais alta do que a média mundial, o que indica haver custo-efetividade para seleção daquelas que devem fazer a antibioticoterapia profilática. É necessária análise mais detalhada de outros fatores relacionados a este protocolo.
